# Making the Most of Adversity: A Fully Remote Ecological Momentary Assessment and Actigraphy Study of Hospital Nurses

**DOI:** 10.1093/geroni/igab046.3294

**Published:** 2021-12-17

**Authors:** Taylor Vigoureux, Christina Mu, Soomi Lee

**Affiliations:** University of South Florida, Tampa, Florida, United States

## Abstract

The COVID-19 pandemic has created challenges and opportunities for research. This is especially true for research on essential workers, such as hospital nurses. In adaptation to the pandemic, the current study aimed to assess the feasibility and acceptability of a fully remote study to collect data on psychological and behavioral measures such as daily stress and sleep, utilizing ecological momentary assessments (EMA) and sleep actigraphy. Our remote study protocol was conducted through a web platform that provided detailed video and written instructions regarding the study and facilitated virtual onboarding meetings with participants. Outpatient day shift nurses (n=86) responded to a background survey, 84 of whom completed 14 days of EMA and sleep actigraphy. Feasibility was assessed by compliance rates to the 14-day study protocol. Acceptability was assessed by analyzing qualitative feedback provided during onboarding meetings (n=82). The compliance rates of EMA (91.8%) and actigraphy (97.9%) were high. The EMA compliance was higher than that from a pre-COVID, non-remote study of inpatient day shift nurses from the same hospital (86.6%, p=.030). Themes from content analysis were mostly positive with 51.2% reporting “easy, clear, simple onboarding process” and 16.3% reporting “helpful website”. Only six participants provided solely negative feedback (e.g., “communication problems” or “technical difficulties/preferences”). Our remote study protocol was feasible and well-accepted by nurses. A similar methodology could be used in studies on broader healthcare workers and those caring for aging populations to better understand their unique challenges and develop effective strategies to help them, both during and after the pandemic.

